# Predictive preoperative clinical score for patients with liver-only oligometastatic colorectal cancer

**DOI:** 10.1016/j.esmoop.2022.100470

**Published:** 2022-04-20

**Authors:** G. Filippini Velázquez, S. Schiele, M. Gerken, S. Neumaier, C. Hackl, P. Mayr, M. Klinkhammer-Schalke, G. Illerhaus, H.J. Schlitt, M. Anthuber, T. Kröncke, H. Messmann, B. Märkl, C. Schmid, M. Trepel, G. Müller, R. Claus, B. Hackanson

**Affiliations:** 1 Comprehensive Cancer Center Augsburg (CCCA), University Medical Center Augsburg, Augsburg, Germany; 2 Faculty of Applied Mathematics and Statistics, University of Augsburg, Augsburg, Germany; 3 Tumor Center Regensburg, Institute for Quality Assurance and Health Service Research, University of Regensburg, Regensburg, Germany; 4 Department of Haematology and Oncology, Katharinen Hospital Stuttgart, Stuttgart, Germany; 5 Department of Surgery, University Medical Center Regensburg, Regensburg, Germany; 6 General Pathology and Molecular Diagnostics, Faculty of Medicine, University of Augsburg, Augsburg, Germany; 7 Faculty of Medicine, University of Freiburg, Freiburg, Germany

**Keywords:** colorectal cancer, oligometastases, clinical score, predictive score, surgical resection, liver metastases, overall survival

## Abstract

**Background:**

Resection of liver metastases from colorectal cancer (CRC) in the oligometastatic stage improves survival and is a potentially curative treatment. Thus, predictive scores that reliably identify those patients who especially benefit from surgery are essential.

**Patients and methods:**

In this multicenter analysis, 512 patients had undergone surgery for liver metastases from CRC. We investigated distinct cancer-specific risk factors that are routinely available in clinical practice and developed a predictive preoperative score using a training cohort (TC), which was thereafter tested in a validation cohort (VC).

**Results:**

Inflammatory response to the tumor, a right-sided primary tumor, multiple liver metastases, and node-positive primary tumor were significant adverse variables for overall survival (OS). Patients were stratified in five groups according to the cumulative score given by the presence of these risk factors. Median OS for patients without risk factors was 133.8 months [95% confidence interval (CI) 81.2-not reached (nr)] in the TC and was not reached in the VC. OS decreased significantly for each subsequent group with increasing number of risk factors. Median OS was significantly shorter (*P* < 0.0001) for patients presenting all four risk factors: 14.3 months (95% CI 10.5 months-nr) in the TC and 16.6 months (95% CI 14.6 months-nr) in the VC.

**Conclusions:**

Including easily obtainable variables, this preoperative score identifies oligometastatic CRC patients with prolonged survival rates that may be cured, and harbors potential to be implemented in daily clinical practice.

## Highlights

• We identified four variables of adverse outcome for patients treated with surgical resection of liver metastases from CRC.
• Adverse variables were inflammatory response to the tumor, multiple metastases, right-sided primary tumor, node-positive primary tumor.
• We developed a preoperative clinical score according to the number of risk factors present.
• Using easily obtainable variables, this score identified patients with oligometastatic CRC with good prognosis.
• Patients without risk factors should strongly be considered for surgical resection of their metastases.

## Introduction

Colorectal cancer (CRC) is the second most frequent cancer in Europe. Almost 50% of patients with CRC will eventually develop metastases, contributing to high mortality rates. About 25% of patients have widespread metastatic disease already at diagnosis, rendering them ineligible for surgical resection in curative intention.^1^ However, resection of liver metastases in limited metastatic stage has significantly improved survival in subsets of these patients and is currently considered a curative treatment approach.^2^, ^3^, ^4^, ^5^, ^6^, ^7^, ^8^

The oligometastatic state of cancer represents an inconsistently defined transition between localized and widespread disease that is characterized by limited number and sites of metastases.^9^ The diagnosis of oligometastatic disease is currently based solely on radiographic imaging.^10^ The term oligometastatic state, first described by Hellman and Weichselbaum,^11^^,^^12^ implies that if the primary tumor site is treated in curative intent and metastatic sites are ablated, long-term disease-free intervals and even cure can potentially be achieved. Thus reliable identification of oligometastatic state patients who will benefit from local therapy using predictive scores is clinically essential.

In a pivotal study, Fong et al.^4^ identified five parameters (nodal status of primary tumor, disease-free interval from diagnosis to discovery of metastases, more than one metastasis, carcinoembryonic antigen level, and tumor size) as significant predictors for overall survival (OS) and established the Clinical Risk Score. Recently, Rees et al.^5^ published the Basingstoke Predictive Index, a score consisting of preoperative and postoperative variables (tumor differentiation grade, extrahepatic metastases, and resection margins in addition to the variables described by Fong et al.^4^). Both scores identified low-risk groups with a median disease-free survival (DFS) of 74 months and a median cancer-specific survival of 7.5 years, respectively. Malik et al.^6^ reported that the presence of a systemic inflammatory response to the tumor (IRT) constitutes an important risk factor for outcome in these patients. These studies were performed before the introduction of antibody-based epidermal growth factor receptor (EGFR)- and vascular endothelial growth factor-directed therapies. Despite these and other previous efforts^3^^,^^7^ to further identify patients with oligometastatic CRC, current clinical scores are still lacking reliability for accurate prediction.^13^ Therefore there is an unmet need to establish scores that better discriminate oligometastatic cases from apparent ones with occult systemic disease before local therapy in curative intent is initiated. Two very recent studies, the ‘Metro ticket’,^14^ which calculates the tumor burden score using size and number of tumors, and the ‘Genetic and Morphological Evaluation (GAME)’ score,^15^ which identified six variables of adverse outcome including the tumor burden score, might be useful strategies in the current era of liver metastases treatment. Although these scores perform well, their applicability is limited because they require information that is not always readily accessible in daily clinical practice.

We conducted a multicenter retrospective analysis of patients after surgical resection of liver metastases from CRC aiming at re-evaluating the known prognostic factor IRT and testing clinically accessible parameters for improving outcome prediction. In addition, we sought to investigate current findings reflecting tumor biology such as the effect of tumor sidedness in the oligometastatic setting. Aiming particularly at clinical applicability, we set up and validated a simple novel preoperative risk model of OS for oligometastatic treatment in CRC to identify patients with favorable prognosis who profit from surgical resection of liver metastases.

## Patients and methods

### Patient cohort and data collection

Clinical and therapeutic data were collected retrospectively and included information available from routine medical records. Only patients with liver metastases treated with surgical resection that were pathologically confirmed as colon adenocarcinoma were included. Patients who died from postoperative complications and not from tumor progression were excluded from analyses, as well as patients with extrahepatic and peritoneal metastases. Only patients with existing preoperative C-reactive protein (CRP) values within a period of 30 days before surgery and without evidence for concurrent infectious complications were included. CRP levels of ≥ 1 mg/dl were considered positive for IRT. Conversely, a CRP value <1 mg/dl was interpreted as absence of IRT.^6^ Molecular genetic analyses of tumor samples included identification of mutations in exons 2, 3, and 4 of the *KRAS* and *NRAS* genes*.* Tumors arising from the ascending colon and the colon transversum were defined as ‘right sided’, whereas all tumors arising from the beginning of the left colon flexure, including descending colon, sigma, and upper rectum, were defined as ‘left sided’. Number of liver metastases, presence of extrahepatic metastases, and lymph node involvement were preoperatively evaluated by computed tomography, magnetic resonance imaging, or ultrasound. These findings were confirmed by pathology and documented in medical records. All patients were initially treated in curative intention for the primary tumor. Perioperative radio/chemotherapy regimens in neoadjuvant or adjuvant intention included 5-fluorouracil or its prodrug capecitabine, alone or in combination with oxaliplatin (FUFOX, FOLFOX, CAPOX), irinotecan (FOLFIRI, Capecitabine/Irinotecan), or a combination of all three substances (FOLFIRINOX/FOLFOXIRI) with or without the anti-VGEF-antibody bevacizumab or an anti-EGFR antibody (cetuximab/panitumumab) according to the tumor board decision. Type, duration, and doses varied according to toxicity, comorbidities, age, and patient’s decision. Informed consent was obtained in accordance with institutional review board policies; data collection and analyses were performed according to the terms of the Declaration of Helsinki.

### Statistical analyses

The entire dataset was divided into a training and a validation set. Patients treated at the University Hospital Augsburg and at 13 peripheral centers, whose data were centralized at the Tumor Center Regensburg, Institute for Quality Assurance and Health Services Research, University of Regensburg, constituted the training cohort (TC; *n* = 282); patients from the University Hospital Regensburg and Katharinen Hospital Stuttgart constituted the validation cohort (VC; *n* = 230). Differences of variable distribution between the two sets were tested with chi-square test for categorical and *t*-test for continuous variables. To enable a non-continuous scoring of age, this variable was split according to the threshold showing the best performance in a univariable regression among all possible values.

The endpoints OS and DFS were defined as time between the date of liver surgery and date of death, and any radiologically confirmed recurrence of the disease, respectively. Patients without recurrence and those alive at last follow-up were censored from the analysis. Cut-off date for survival analyses was 31 December 2019. Follow-up was calculated by the reverse Kaplan–Meier method.

Based on the TC, a separate univariable proportional hazards model was fitted for both outcomes and each clinical variable to determine appropriate variables for a multivariable model. Variables showing *P* values <0.15 in univariable analyses were selected for a multivariable proportional hazard model with *P* value based backward selection. We tested Cox model assumptions of proportional hazards via Schoenfeld residuals. Hazard ratios (HRs) and the corresponding 95% confidence intervals (CIs) were calculated for each final model. We selected variables remaining in both final models and assigned them the rounded quotient of their HR and the smallest HR of the model as score. Each variable added 1 point to the score when present. Patients in the TC were stratified into five different risk groups according to the resulting cumulative score. For each risk group, OS and DFS was estimated by Kaplan–Meier curves using the log-rank test for significance. This score was thereafter validated in an independent VC. Finally, we compared the results of the novel score with the score of Malik et al.^6^ regarding risk stratification*.* All tests were performed two sided on a significance level of 5% using statistical computing program R version 4.0.2 (R Foundation, Vienna, Austria) and IBM SPSS Statistic 25 (New York, NY).

## Results

**Figure 1:**
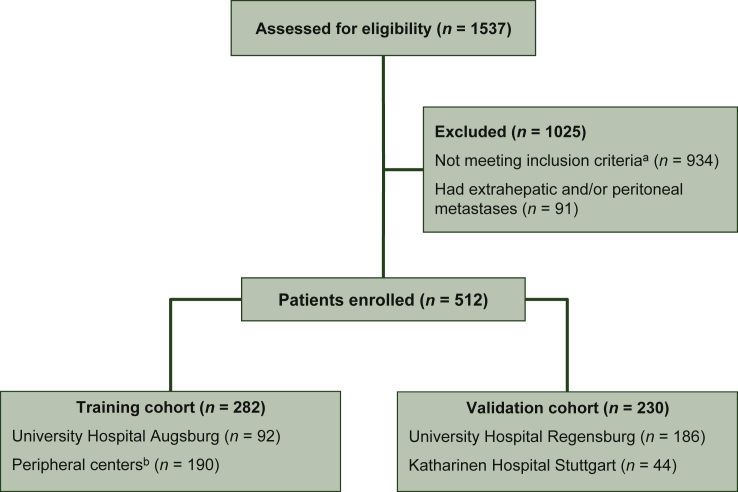
Consort diagram of patients enrolled in the study. CRC, colorectal cancer; CRP, C-reactive protein; FAP, familial adenomatous polyposis; GIST, gastrointestinal stromal tumor; NET, neuroendocrine tumor; RFA, radiofrequency ablation; SCC, squamous cell carcinoma. ^a^Patients with liver metastases from CRC not treated in curative intention with surgical resection of liver metastases (e.g. only RFA, only chemotherapy), documented to die from postoperative complications (not from tumor progression), postoperative histological diagnosis other than adenocarcinoma of the colon (NET, GIST, SCC), with FAP, incomplete data for CRP values, CRP values older than 30 days before liver surgery, concurrent infectious disease or inflammation due to complications of tumor progression, extrahepatic metastases, diffuse peritoneal metastases. ^b^Tumor Center Regensburg, University of Regensburg.

Clinical records of 1537 patients were assessed for eligibility, 1025 patients did not meet the inclusion criteria and were excluded from analyses. The remaining 512 patients with metastatic CRC who underwent surgical resection of *de novo* liver metastases between 2006 and 2016 at 16 different hospitals in Germany were included in this analysis. A total of 322 (62.9%) patients were treated at 3 high-volume centers and 190 were treated at 13 peripheral clinics (Figure 1). Thirty (5.9%) patients received combined surgery and thermoablation. There was no significant difference between the TC and VC regarding the presence of IRT, right-sided primary tumor, node-positive primary tumor, positive resection margins, sex, *KRAS* mutation status, synchronous disease, and perioperative chemotherapy.

The TC included more patients with age >72 years at the time of surgery (*P* = 0.044), and the VC comprised more patients with multiple liver metastases (*P* = 0.041). In the TC, median age at time of surgery was 68 years; 86 (30.5%) patients were aged >72 years. A total of 136 (48.2%) patients had multiple liver metastases [median: 1 (r: 1–9)]. In the VC, median age was 65 years and 51 (22.2%) patients were older than 72 years. A total of 131 (57.0%) patients had multiple liver metastases [median: 2 (r: 1–14)]. Overall, 249 (81.6%) patients with synchronous, and 97 (46.9%) with metachronous disease received preoperative or postoperative chemotherapy. Details about perioperative chemotherapy protocols are given in the Supplementary Results, available at https://doi.org/10.1016/j.esmoop.2022.100470. section. A detailed list of patient characteristics is presented in Table 1.

**Table 1:** Patient characteristics

Variables		Training (*n* = 282)	Validation (*n* = 230)	Total (%), (*n* = 512)	*P* value
Sex, *n* (%)	Female	92 (32.6)	67 (29.1)	159 (31.1)	0.451
	Male	190 (67.4)	163 (70.9)	353 (68.9)	
Median age at the time of surgery, years (range)		68 (31-89)	65 (27-88)	66 (27-89)	<0.001
Age at the time of surgery, *n* (%)	<72 years	196 (69.5)	179 (77.8)	375 (73.2)	0.044
	>72 years	86 (30.5)	51 (22.2)	137 (26.8)	
IRT, *n* (%)	No IRT	217 (77.0)	181 (78.7)	398 (77.7)	0.715
	IRT	65 (23.0)	49 (21.3)	114 (22.3)	
Primary tumor side, *n* (%)	Left	215 (76.2)	164 (71.3)	379 (74.0)	0.244
	Right	67 (23.8)	66 (28.7)	133 (26.0)	
Median number of liver metastases (range)		1 (1-9)	2 (1-14)	2 (1-14)	0.009
Solitary versus multiple liver metastases, *n* (%)	Solitary	146 (51.8)	96 (41.7)	242 (47.3)	0.041
	Multiple	136 (48.2)	131 (57.0)	267 (52.1)	
	Missing data	0 (0.0)	3 (1.3)	3 (0.6)	
Node-positive primary tumor, *n* (%)	Negative	94 (33.3)	74 (32.2)	168 (32.8)	0.816
	Positive	179 (63.5)	150 (65.2)	329 (64.3)	
	Missing data	9 (3.2)	6 (2.6)	15 (2.9)	
Synchronous versus metachronous disease, *n* (%)	Metachronous	110 (39.0)	97 (42.2)	207 (40.4)	0.525
	Synchronous	172 (61.0)	133 (57.8)	305 (59.6)	
*KRAS*, *n* (%)	Wildtype	91 (32.3)	45 (19.6)	136 (26.6)	0.875
	Mutated	44 (15.6)	24 (10.4)	68 (13.3)	
	Missing data	147 (52.1)	161 (70.0)	308 (60.2)	
Ctx, *n* (%) Preoperative Postoperative Preoperative or postoperative (perioperative Ctx)	Yes	88 (31.2)	81 (35.2)	169 (33.0)	0.247 0.641 0.187
	No	191 (67.7)	139 (60.4)	330 (64.5)	
	Missing data	3 (1.1)	10 (4.3)	13 (2.5)	
	Yes	142 (50.4)	117 (50.9)	259 (50.6)	
	No	120 (42.6)	90 (39.1)	210 (41)	
	Missing data	20 (7.1)	23 (10)	43 (8.4)	
	Yes (pre/post or both)	184 (65.2)	162 (70.4)	346 (67.6)	
	No Ctx (pre and post)	86 (30.5)	55 (23.9)	141 (27.5)	
	Missing data	12 (4.3)	13 (5.5)	25 (4.9)	
Resection margin status, *n* (%)	R0	227 (80.5)	184 (80.0)	411 (80.3)	0.336
	R1	25 (8.9)	28 (12.2)	53 (10.4)	
	Missing data	30 (10.6)	18 (7.8)	48 (9.4)	

Note: variables with significant differences between the training and validation cohorts were age at the time of surgery and number of liver metastases.

CTx, chemotherapy; IRT, inflammatory response to the tumor.

The median follow-up for the entire cohort was 81.2 months (TC: 83.2 months; VC: 70.3 months), and the median OS and DFS for the entire cohort was 60.4 months (95% CI 52.2-68.5 months) and 17.0 months (95% CI 14.3-19.8 months), respectively.

### Univariable analyses

The selected clinical parameters were tested in univariable analyses for DFS and OS in the TC. Results are shown in Table 2.

**Table 2:** Univariable analysis of overall survival and disease-free survival

Variables	Significance (log rank)	
	Overall survival	Disease-free survival
Male sex	0.225	0.016
Age at the time of surgery (>72 years)	<0.001	0.400
Inflammatory response to tumor	<0.001	<0.001
Right-sided primary tumor	0.009	0.002
Solitary versus multiple metastases	<0.001	0.005
Node-positive primary tumor	0.021	0.149
Synchronous disease	0.014	0.143
Perioperative chemotherapy	0.558	0.398
Resection margin status (R1)	0.082	0.003
*KRAS* mutated	0.055	0.016

Note: preoperative variables with *P* value <0.15 were included in a multivariable model.

The impact of the number of resected metastases on outcome was evaluated using the presence of one liver metastasis as reference for the HR, with >1 liver metastases resulting in a significantly increased risk for reduced DFS (HR 1.5) and OS (HR 2.1), which remained constant for >2 metastases (HR 1.4 for DFS; HR 2.1 for OS). Thus, only ‘solitary versus multiple liver metastases’ (number of metastases >1) was considered as variable for number of metastases.

We observed that ‘age at the time of surgery’ as a continuous variable showed increasing significance with increasing age for the endpoint OS. For univariable analyses, we tested age as categorical variable and determined the cut-off at which age had the lowest *P* value. Age >72 years resulted in a significant independent risk factor for OS (*P* < 0.001; HR 1.7) but not for DFS. The HR of age increased more than twofold (HR 2.9) for OS in patients aged >80 years.

Two variables that reflect biological features of the disease (primary tumor side and IRT) had significant impact on outcome. Median OS for patients with left-sided primary tumor was 65.2 months (95% CI 55.6-74.8 months) and 41.1 months (95% CI 25.8-56.4 months) for right-sided primary tumor patients (*P* = 0.009 log rank). Patients with left-sided primary tumor had a median DFS of 19.7 months (95% CI 15.5-23.9 months), whereas those with right-sided primary tumor had a median DFS of 10.8 months (95% CI 5.9-15.6 months; *P* = 0.002 log rank).

OS for patients without IRT was 72.7 months (95% CI 63.6-81.7 months). By contrast, patients with IRT had a median OS of 28.2 months (95% CI 18.4-38.0 months; *P* < 0.0001 log rank). Patients without IRT had a median DFS of 20.4 months (95% CI 16.4-24.3 months), whereas presence of IRT resulted in a median DFS of 10.8 months (95% CI 8.6-13.0 months; *P* < 0.0001 log rank).

### Multivariable analyses

For OS, five variables showed significance with a *P* value <0.05: IRT (*P* < 0.001; HR 1.92; 95% CI 1.35-2.75), right-sided primary tumor (*P* = 0.008; HR 1.63; 95% CI 1.14-2.34), multiple liver metastases (*P* < 0.001; HR 1.75; 95% CI 1.27-2.42), node-positive primary tumor (*P* = 0.026; HR 1.49; 95% CI 1.05-2.13), and age >72 years at the time of surgery (*P* = 0.001; HR 1.72; 95% CI 1.24-2.44).

For DFS, four variables had a *P* value <0.05: IRT (*P* = 0.002; HR 1.74; 95% CI 1.23-2.47), right-sided primary tumor (*P* = 0.014; HR 1.56; 95% CI 1.09-2.21), multiple liver metastases (*P* = 0.016; HR 1.46; 95% CI 1.07-1.98), and male sex (*P* = 0.035; HR 1.44; 95% CI 1.03-2.03; Supplementary Table S1, available at https://doi.org/10.1016/j.esmoop.2022.100470).

### Predictive score for patients undergoing local treatment in oligometastatic CRC

Four variables [IRT (HR 1.92), right-sided primary tumor (HR 1.63), multiple liver metastases (HR 1.75), and node-positive primary tumor (HR 1.49)] that were significant for OS in multivariable analyses were selected and integrated into a predictive score. Age, although significant, was not included in the score as it was not considered a cancer-specific risk factor. All variables that were significant for OS, with the exception of node-positive primary tumor, were also significant for DFS. We focused the survival analyses on OS. Kaplan–Meier survival analyses of DFS are presented separately in Supplementary Figure S1, available at https://doi.org/10.1016/j.esmoop.2022.100470. Each variable added 1 point to the score when present. Differential weighting of single factors was not considered necessary, because all of them shared comparable HRs between 1.49 and 1.92.

**Figure 2:**
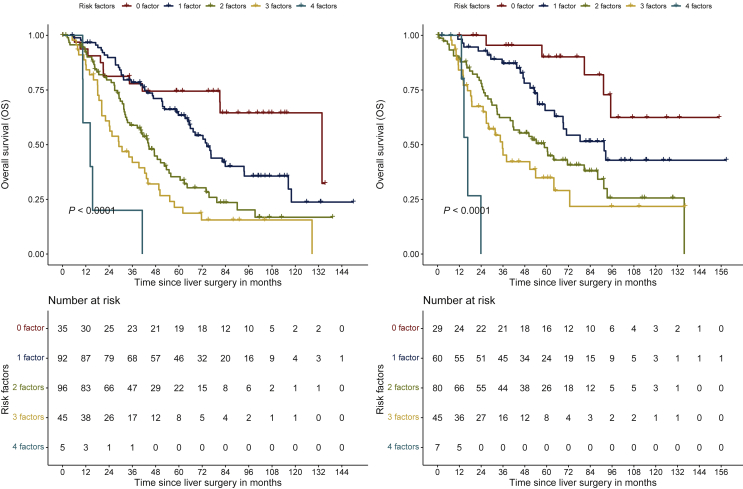
**Stratification of patients according to the number of risk factors. Overall survival for the training (left) and validation cohorts (right) are shown**.

This model of patient stratification according to the cumulative number of their positive risk factors resulted in five risk groups (0, 1, 2, 3, and 4 risk factors). Kaplan–Meier survival analyses based on our score significantly distinguished OS (*P* < 0.0001) between all risk groups in both cohorts (TC and VC). The median OS for the lowest risk group (0 risk factors) in the TC was 133.8 months [95% CI 81.2 months-not reached (nr)], and was not reached in the VC (95% CI 95.2 months-nr). The highest risk group (all four risk factors) had a median OS of 14.3 months (95% CI 10.5 months-nr) in the TC and 16.6 months (95% CI 14.6 months-nr) in the VC (Figure 2, Table 3). The impact of the score on DFS is described in Supplementary Results and Table S2, available at https://doi.org/10.1016/j.esmoop.2022.100470.

**Table 3:** Predictive preoperative score for oligometastatic colorectal cancer

Risk group (definition)	Number of patients (training/validation)	Median OS in months (*P* < 0.0001)	
		Training (95% CI)	Validation (95% CI)
0 risk factors	35/29	133.8 (81.2-nr)	nr (95.2-nr)
1 risk factor	92/60	74.4 (65.3-93.7)	91.6 (69.0-nr)
2 risk factors	96/80	44.4 (34.7-54.9)	58.8 (41.5-91.4)
3 risk factors	45/45	29.0 (22.1-44.0)	35.7 (26.8-72.7)
4 risk factors	5/7	14.3 (10.5-nr)	16.6 (14.6-nr)

CI, confidence interval; nr, not reached; OS, overall survival. Risk factors; Inflammatory response to the tumor (IRT), right-sided primary tumor, multiple liver metastases, node-positive primary tumor.

To compare our score with the score from Malik et al.,^6^ we stratified patients of our VC according to the presence of IRT and number of metastases (>8) into three risk groups (score 0, 1, and 2) as previously described by Malik et al.^6^ OS for the lowest risk group (absence of IRT and <8 metastases) was 67.3 months (95% CI 57.0-77.6 months). Thus, the direct comparison of both scores based on our VC identified a group of low-risk patients with significantly increased OS when applying our novel scoring system. The presence of eight metastases was rarely observed; therefore risk group patients with score 2 were rare in our cohort.

For performance calculation, we dichotomized our score according to the risk groups and, to overcome the problem of patients with censored survival, excluded all patients censored until 12 months of follow-up. For OS, the Harrel’s c-index of our score was 0.676 in comparison to a c-index of 0.616 for the Malik score when applied to our cohort.

## Discussion

To define the oligometastatic stage of cancer and predict which patients will benefit most likely from surgical resection of metastases, the determination of predictive biomarkers is essential. We identified four preoperative cancer-specific risk factors for survival in patients that had undergone surgical resection of liver metastases from CRC and developed a predictive preoperative clinical score. Two risk factors, namely, ‘presence of an IRT’ and ‘right-sided primary tumor’, are linked to disease biology while ‘solitary versus multiple liver metastases’ and ‘node-positive primary tumor’ additionally reflect the dynamics of the disease and evolution in time. Although age at the time of liver surgery was significant for OS in multivariable analyses, this variable was not included into the score because it does not represent a cancer-specific risk factor. In addition, the influence of age could reduce the clinical relevance and validity of the score due to differences between chronological and biological age and thus bias the applicability of the results. In our study, we investigated the impact of the listed clinical variables on OS and DFS but mainly focused on OS. One variable (node-positive primary tumor) that was significant for OS did not reach significance for DFS, which might be due to a type II error.

We assume that the ability of the tumor to invade and metastasize is dependent not only on the innate tumor biology, but also on the diversity of the tumor microenvironment and function of their stroma cells. The interaction between the tumor and the host by means of interleukins (ILs) and cytokines generates a chronic inflammation, which induces genomic instability and fosters tumor evolution. This results in an attractive environment for tumor growth, invasive potential, and angiogenesis, favoring neoplastic spread and metastasis,^16^, ^17^, ^18^, ^19^, ^20^ whereas high levels of T-cell infiltration are predictive of better outcome in CRC.^21^^,^^22^

We hypothesize, that the proinflammatory immune response to the tumor causes a systemic inflammatory response that can be detected by measuring serum CRP. CRP is an acute-phase protein and sensitive systemic marker of tissue damage, produced in response to IL-1 and IL-6. Its plasma concentration increases significantly in inflammatory states and drops rapidly again when the stimulus ceases. These and other biological features make CRP an attractive potential tumor marker.^23^^,^^24^ There is also clinical evidence of the prognostic value of CRP in gastrointestinal malignancies.^25^, ^26^, ^27^, ^28^, ^29^ Other potential markers of IRT are the neutrophil-to-lymphocytes ratio, cytokines (tumor necrosis factor-α, IL-6, IL-1B, CCL2), or the quantification of lymphocytes subpopulations in the tumor (Immunoscore).^30^ We chose CRP for reasons of practicality and ease of availability in medical records, basing our cut-off value of 1 mg/dl on a previous study with a similar scientific question^6^ and other CRC-related studies that also applied this cut-off value.^27^^,^^28^

Primary tumors arising from the left and right sides of the colon have a different developmental origin resulting in distinct clinical and molecular characteristics, incidence rates, and marked differences in the microbiome diversity. Right-sided tumors were more commonly associated with *RAS* and *BRAF* mutations, whereas higher EGFR expression and *HER2/neu* amplification were more prevalent in left-sided CRC.^31^, ^32^, ^33^ However, mutant *KRAS* was not a significant risk factor in our analysis, most likely because of incomplete available data in our cohort. Several studies have investigated the predictive effect of primary tumor location among all stages of CRC, concluding that primary location is an important risk factor.^34^, ^35^, ^36^, ^37^, ^38^

Our clinical score identified a group of patients without risk factors and prolonged survival rates after surgical treatment of liver metastases that may accurately represent the genuine and potentially curable oligometastatic stage of CRC. The score also identified a subsequent risk group carrying one risk factor with a prolonged survival despite experiencing recurrence of metastases. This group of patients may harbor biological features that translate clinically into ‘oligorecurrence’ or ‘oligoprogression’, scenarios that imply a noncurable stage of tumor disease of limited metastatic potential, making them likewise suitable for surgical resection of metastases with or without additional systemic therapy achieving prolonged disease-free intervals.^10^^,^^39^

By contrast, the group of patients carrying all risk factors had a significantly shorter OS (*P* < 0.0001). This group of patients apparently have tumors that represent a stage with biological and clinical characteristics beyond the oligometastatic state of cancer. Depriving this group of patients from the potential benefits of surgical resection is questionable. Prospective trials addressing the role of additionally intensive perioperative chemotherapy or alternative less invasive ablative technics in these ‘very-high-risk’ patients could provide relevant information on this matter.^40^

We systematically compared our clinical score with the score of Malik et al.,^6^ which includes IRT and number of metastases as risk factors. While the performance of our score was noninferior in a direct comparison, the additional risk factors integrated in our score conferred the particular advantage of identifying a subgroup of IRT-negative patients with remarkably longer OS.

Dupré et al.^41^ published the ‘Liverpool score’ for CRC liver metastases treated in curative intention. Four of the six preoperative variables of significance for OS in their score, which stratifies patients into two risk groups, are also present in our score. In their case, IRT was defined by the neutrophil-to-lymphocytes ratio. The similarity between both scores emphasizes the relevance of these risk factors for prediction in the treatment of CRC liver metastases. Nonetheless, we see the advantage of our score in the ability to identify well-defined risk groups using less variables and thus easier applicability in clinical settings.

Moreover, two very recent studies (the ‘Metro ticket’^14^ and the ‘GAME’ score^15^) nicely demonstrated useful strategies in the current era of liver metastases treatment. However, the applicability of these well-performing scores may be limited because some of the variables are not always easily accessible in daily clinical practice.

Recently, Pitroda et al.,^42^ in a fascinating work integrating RNA-based molecular subtyping and Fong’s Clinical Risk Score,^4^ identified a subgroup of patients with metastatic CRC that clinically performed in concordance with a potentially curable oligometastatic state. Furthermore, supporting the findings of Galon et al.,^21^ these tumors were distinguished by gene signatures that correlated histologically with high levels of T-cell infiltrations.^43^

Here, we identified and validated four clinical risk factors in the context of oligometastatic CRC, two of which support biological aspects implicated in the origin of oligometastases. In fact, considering IRT as significant predictive factor, we believe that our study, based on the findings of Malik et al.,^6^ represents an important step towards finding specific biomarkers that precisely help to define this state of disease.

We emphasize on the practicability of our score, because it contains variables that are routinely available in clinical practice, on its robustness, relying on the multicenter analysis including high- and low-volume centers, as well as the successful reproduction of the score among heterogenic cohorts. Limiting factors are the retrospective nature of this study, the heterogeneity in patient’s characteristics, and the number of cases censored from the analysis. It is also presumable that the sensitivity of radiographic imaging has improved over time in the period of patient enrollment. Although we used a well-comprehensible and previously applied CRP value cut-off for IRT definition, this value has been described as variable in other studies^29^ and could be different in each patient. In addition, CRP is a nonspecific IRT marker, and its value may be altered by other causes of inflammation (chemotherapy, obesity, smoking, coronary heart disease)^29^ for which this study was not adjusted. A final potential limitation is the impact of chemotherapy on relapse incidence and outcome. Perioperative chemotherapy regimens were heterogeneous and varied according to synchronous/metachronous disease, age, performance status, comorbidities, and decision of the patients (Supplemental Results, available at https://doi.org/10.1016/j.esmoop.2022.100470). In addition, patients with a higher risk of disease progression defined by nodal status, tumor size, and number of metastases were likely to receive more intensive chemotherapy.

Taken together, our preoperative clinical score includes variables that reflect biological aspects of the disease and are easily obtainable from medical records. The score is easy to apply and shows potential to be implemented in daily clinical practice for the identification of patients in the oligometastatic state of CRC that considerably profit from local treatment.
