# Supplementary material for: Predictive preoperative clinical score for patients with liver-only oligometastatic colorectal cancer
Source: ESMO Open. 2022 Apr 20;7(3):100470. doi: 10.1016/j.esmoop.2022.100470 (PMC9271475; doi:10.1016/j.esmoop.2022.100470)
Supplement: Supplementary Material [file mmc1.docx]

**Supplementary results**

***Chemotherapy***

Overall, 249 (81,6%) patients with synchronous, and 97 (46,9%) with metachronous disease received chemotherapy (at least preoperative or postoperative). Preoperative, the most common used chemotherapy protocol was FOLFOX (47,9%), followed by FOLFIRI (27,2%), 5-FU (15,4%), Capecitabine (3%), Capox (1,8%), FOLFOXFIRI (1,8%), FUFOX (1,2%), FOLFIRINOX, Irinotecan, Capecitabine/ Irinotecan (0,6%, respectively). Postoperative protocols used were FOLFOX (57,8%), FOLFIRI, Capecitabine (12,9%, respectively), 5-FU (10,3%), CAPOX (2,6%), FUFOX (2,2%), Capecitabine/ Irinotecan (0,9%), FOLFOXFIRI (0,4%). 100 (19,5%) received an anti-EGFR or anti-VGEF monoclonal antibody. Reasons for not applying chemotherapy were in decreasing order of frequency: Tumor board decision, patient´s desire, age, comorbidity, limiting toxicity. Common causes for not receiving a chemotherapy in the synchronous situation were simultaneous surgical resection of the primary tumor and metastases and a “liver first” approach, with early progression of disease or operative complications with subsequent delay with the beginning of chemotherapy before progression of the disease. The most common reason in the metachronous situation was the tumor board decision.

**Predictive score for patients undergoing local treatment in oligometastatic CRC**

***Disease free survival***

The group without risk factors did not reach median DFS (CI 95%: 22.0-nr) in the TC (n=35) and had a median DFS of 80.2 months (CI 95%: 60.0–nr) in the VC (n=29). DFS was significantly shorter [p<0.0001 (TC), p=0,00051 (VC)] for the subsequent groups with increasing number of risk factors. The highest risk group, presenting with all four risk factors had a median DFS of 9.3 months (CI 95%: 4.2-nr) in the TC (n=5) and 3.7 months (CI 95%: 2.9.-nr) in the VC (n=7) **(Supp. Figure 1, Supp. Table 2).**
