# Supplementary material for: Predictive preoperative clinical score for patients with liver-only oligometastatic colorectal cancer
Source: ESMO Open. 2022 Apr 20;7(3):100470. doi: 10.1016/j.esmoop.2022.100470 (PMC9271475; doi:10.1016/j.esmoop.2022.100470)
Supplement: Supplementary Table S1 and S2 [file mmc3.docx]

**Supplemental Tables**

| **Supp. Table 1 - Multivariable analyses of overall survival and disease-free survival** | | | | |
| --- | --- | --- | --- | --- |
| **Variables** | **Overall survival** | | **Disease-free survival** | |
|  | Significance | Hazard ratio  (CI 95%) | Significance | Hazard ratio  (CI 95%) |
| **Inflammatory response to tumor** | **<0.001** | **1.92 (1.35-2.75)** | 0.002 | 1.74 (1.23-2.47) |
| **Right-sided primary tumor** | **0.008** | **1.63 (1.14-2.34)** | 0.014 | 1.56 (1.09-2.21) |
| **Solitary vs multiple liver metastases** | **<0.001** | **1.75 (1.27-2.42)** | 0.016 | 1.46 (1.07-1.98) |
| **Node positive primary tumor** | **0.026** | **1.49 (1.05-2.13)** | --- | --- |
| Age at time of therapy (> 72y) | 0.001 | 1.72 (1.24-2.44) | --- | --- |
| Male sex | --- | --- | 0.035 | 1.44 (1.03-2.03) |

**Note: Inflammatory response to tumor, right-sided primary tumor, multiple metastases (>1) and node positive primary tumor were variables that composed the preoperative risk score.**

| **Supp. Table 2 - Predictive preoperative score for oligometastatic colorectal cancer** | | | |
| --- | --- | --- | --- |
| **Risk group**  **(Definition)** | **Number of patients**  **(Training / Validation)** | **Median DFS (months)**  **(p <0.0001)** | |
|  |  | **Training**  **(CI 95%)** | **Validation**  **(CI 95%)** |
| **0 risk factors** | 35 / 29 | Not reached (22.1-nr) | 80.2 (60.0-nr) |
| **1 risk factor** | 92 / 60 | 21.7 (15.1-37.2) | 29.7 (15.9-68.4) |
| **2 risk factors** | 96 / 80 | 12.4 (10.1-20.2) | 21.5 (10.0-35.2) |
| **3 risk factors** | 45 / 45 | 15.0 (5.3-26.7) | 10.7 (6.7-18.7) |
| **4 risk factors** | 5 / 7 | 9.3 (4.2-nr) | 3.7 (2.9-nr) |
