# Supplementary material for: Predictive preoperative clinical score for patients with liver-only oligometastatic colorectal cancer
Source: ESMO Open. 2022 Apr 20;7(3):100470. doi: 10.1016/j.esmoop.2022.100470 (PMC9271475; doi:10.1016/j.esmoop.2022.100470)
Supplement: Supplementary Figure 1 [file mmc2.pdf]

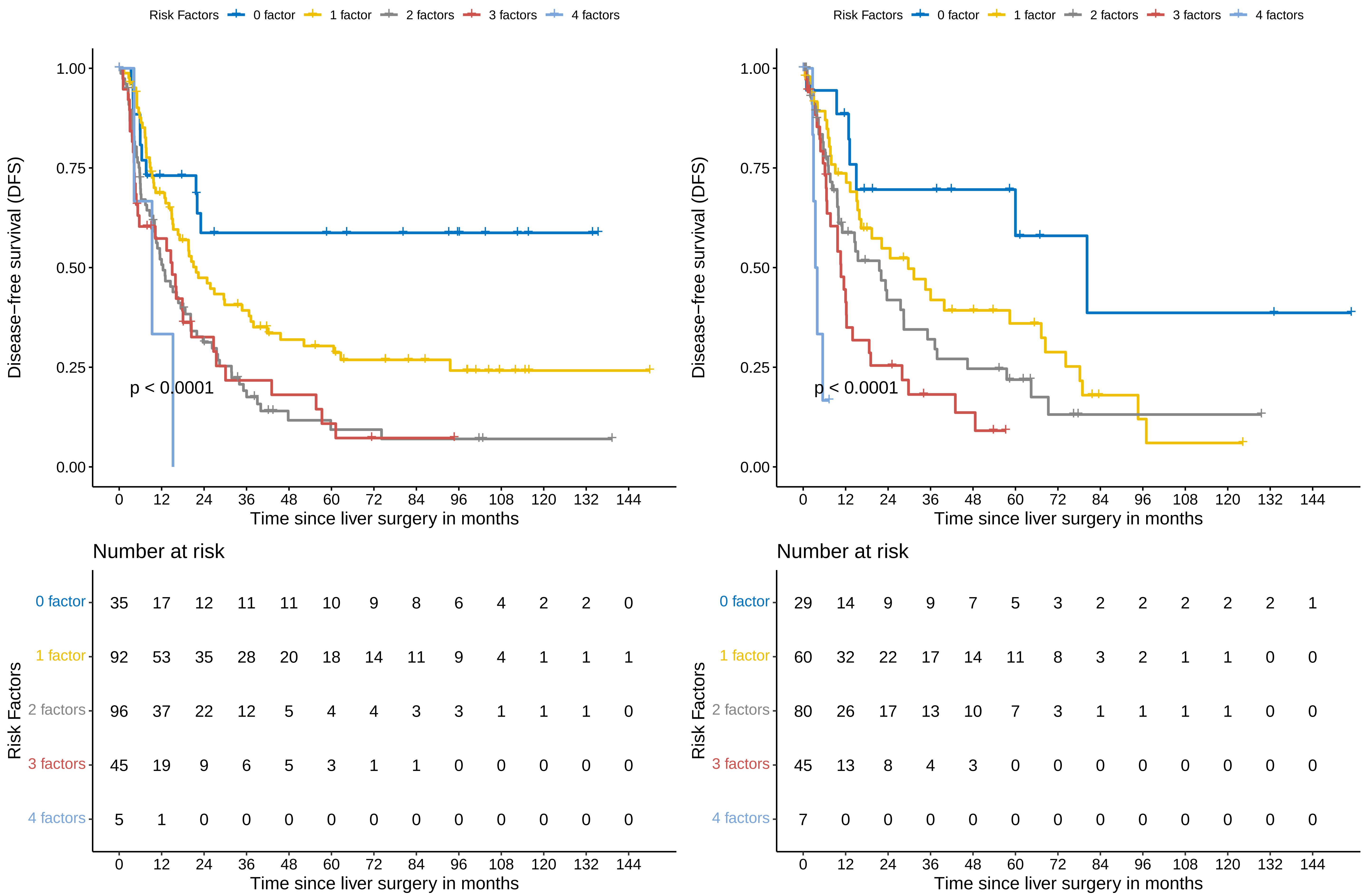
Risk Factors 0 factor 1 factor 2 factors 3 factors 4 factors
Disease−free survival (DFS)
1.00
0.75
0.50
0.25
0.00
p < 0.0001
0 12 24 36 48 60 72 84 96 108 120 132 144
Time since liver surgery in months
Number at risk
Risk Factors
0 factor 35 17 12 11 11 10 9 8 6 4 2 2 0
1 factor 92 53 35 28 20 18 14 11 9 4 1 1 1
2 factors 96 37 22 12 5 4 4 3 3 1 1 1 0
3 factors 45 19 9 6 5 3 1 1 0 0 0 0 0
4 factors 5 1 0 0 0 0 0 0 0 0 0 0 0
0 12 24 36 48 60 72 84 96 108 120 132 144
Time since liver surgery in months
Risk Factors 0 factor 1 factor 2 factors 3 factors 4 factors
Disease−free survival (DFS)
1.00
0.75
0.50
0.25
0.00
p < 0.0001
0 12 24 36 48 60 72 84 96 108 120 132 144
Time since liver surgery in months
Number at risk
Risk Factors
0 factor 29 14 9 9 7 5 3 2 2 2 2 2 1
1 factor 60 32 22 17 14 11 8 3 2 1 1 0 0
2 factors 80 26 17 13 10 7 3 1 1 1 1 0 0
3 factors 45 13 8 4 3 0 0 0 0 0 0 0 0
4 factors 7 0 0 0 0 0 0 0 0 0 0 0 0
0 12 24 36 48 60 72 84 96 108 120 132 144
Time since liver surgery in months
